# Association between nursing work environment and decision fatigue among clinical nurses: a moderated mediation analysis on the roles of job stress and resilience

**DOI:** 10.3389/fpsyg.2025.1676964

**Published:** 2026-01-05

**Authors:** ManZhi Gao, JieFen Ou, HuiXia Cao, FuLu Lv

**Affiliations:** 1 Department of Nursing, The Sixth Affiliated Hospital, School of Medicine, South China University of Technology, Foshan, China; 2 School of Nursing, Guangdong Pharmaceutical University, Guangzhou, China

**Keywords:** decision fatigue, moderated mediation model, clinical nurses, job stress, resilience, nursing work environment

## Abstract

**Background:**

Decision fatigue significantly impairs nurses’ clinical judgment and threatens patient safety. Although the nursing work environment is a recognized contributor, the underlying psychological mechanisms, particularly the roles of job stress and resilience, remain inadequately explored.

**Objective:**

This study aimed to test the relationship between nursing work environment, job stress and decision fatigue among clinical nurses. The study also examined the moderation role of resilience on the relationship between nursing work environment and job stress.

**Methods:**

A cross-sectional study was conducted between May 2023 and July 2023 among clinical nurses in four public hospitals in Guangdong, China. A convenience sampling method was used to collect questionnaire data from 812 clinical nurses. Data analysis was performed by SPSS 27.0, while PROCESS macro v3.5 was used to test the moderated mediation model.

**Results:**

Nursing work environment mediated by job stress had a significant positive predictive effect on decision fatigue. Resilience played a negative moderating role in the relationship between nursing work environment and job stress. For nurses with low resilience, nursing work environment had a greater impact on job stress.

**Conclusion:**

This study highlights the relationship between the nursing work environment and decision fatigue, and how job stress can be mediated and moderated by resilience.

## 1 Introduction

With advancements in medical technology and increasingly diverse patient needs, nurses are facing heavier workloads and more complex clinical decision-making demands. The increasing demand for nursing services, driven by population aging and limited staffing, has led to heavier workloads and heightened stress within the nursing profession, further contributing to burnout and turnover (Ren et al., 2024). Due to the unique nature of nursing work, which involves a high workload, risks, and a complex, ever changing environment, nurses are required to make a multitude of decisions daily, each decision involves a cognitive process of assessment, diagnosis, prediction, and decision-making (Pignatiello et al., 2022; Schweitzer et al., 2023). Prolonged work under such high-intensity conditions can deplete cognitive and emotional resources, particularly when facing complex decision making dilemmas, leading to a state known as decision fatigue (Fernández-Miranda et al., 2023; Dong et al., 2024).

Decision fatigue is defined as the deterioration of decision-making ability and self-control caused by repeated engagement in decision-making tasks (Hickman et al., 2018; Pignatiello et al., 2020). Although conceptually related, decision fatigue differs from general fatigue and self-depletion. Fatigue typically reflects a broad decline in physical and psychological functioning, such as physical exhaustion, reduced attention, and emotional burnout (Bell et al., 2023). In contrast, self-depletion refers to the exhaustion of core executive resources, including sustained attention, impulse control, and emotion regulation. Decision fatigue, therefore, represents a more specific form of self-depletion that primarily impairs decision-related cognitive processes (Baumeister et al., 2018; Pignatiello et al., 2020). Individuals experiencing decision fatigue often exhibit avoidance, procrastination, and impulsive behaviors, as well as reduced executive functioning and reasoning abilities (Rao and Nyquist, 2018; Zhu et al., 2021). It is also characterized by compromised judgment and a greater tendency toward passive or overly conservative decisions. Studies suggest that approximately 29% of nurses experience decision fatigue (Scott et al., 2014), which can undermine both patient safety and care quality. In clinical practice, nurses are frequently required to process large volumes of complex information under time pressure. Prolonged exposure to such cognitive demands may progressively diminish their ability to receive, analyze, and recall information, ultimately leading to decision fatigue (Ayres et al., 2021). Decision fatigue has been shown to impair adherence to clinical protocols and reduce both work efficiency and quality of care (Wilby and Paravattil, 2021; Blissett et al., 2021). Therefore, identifying the factors contributing to nurses’ decision fatigue and clarifying the underlying mechanisms is crucial for improving clinical decision-making and enhancing patient outcomes.

The nursing work environment significantly influences nurses’ psychological well-being and job performance (Kohnen et al., 2023). Such adverse environments, characterized by excessive workload, poor staffing, and limited support, increase stress and undermine decision-making (Pignatiello et al., 2022; Dong et al., 2024). Further corroborating the impact of work-related stressors, a study of Emirati women healthcare workers revealed high levels of anxiety, tension, and work-life balance difficulties, findings that were significantly linked to work-related variables such as shift times and years of experience (Al-Akashee et al., 2024). Consequently, prolonged job stress and repetitive decision-making can lead to cognitive overload, thereby impairing clinical judgment (Persson et al., 2019). In this context, resilience defined as the ability to adapt positively to adversity, plays a crucial role in mitigating these effects. Evidence suggests that resilience buffers the impact of stressors on psychological well-being and reduces turnover intentions (Delgado et al., 2021; Gündüz et al., 2024). A resilient nurse may therefore be better equipped to maintain decision quality despite repeated cognitive demands, potentially moderating the relationship between nursing work environment, job stress, and decision fatigue.

In summary, the nursing work environment is a crucial determinant of decision fatigue. However, existing studies have not been completely clear about the intrinsic mechanisms particularly the mediating role of job stress and the moderating influence of resilience. Grounded in the Job Demands–Resources (JD-R) theory and Self-Control Theory (SCT), this study investigates how the nursing work environment affects decision fatigue through job stress, and how resilience moderates this pathway. The findings aim to inform targeted interventions to alleviate decision fatigue, thereby enhancing nursing well-being and patient care quality.

### 1.1 The relationship between nursing work environment and decision fatigue

The nursing work environment refers to the sum of various elements that directly or indirectly influence the nursing system (Lee and Scott, 2018). It is a crucial factor affecting decision fatigue (Pignatiello et al., 2022). Research has shown that a positive work environment, by providing adequate resources (such as organizational support and a supportive interpersonal atmosphere), can effectively reduce nurses’ cognitive load, thereby improving decision quality and work efficiency (Falguera et al., 2021). Consistent with this perspective, Cai et al. (2024) explored the impact of employee assistance programs on job performance and revealed that the psychological contract and perceived organizational support serve as pivotal mechanisms through which organizational resources translate into enhanced performance and reduced psychological strain among employees. Adequate work resources are essential for optimizing the decision-making process and enhancing overall performance, particularly in terms of social support factors within the work environment (such as relationships with colleagues and supervisors), which play a vital role in alleviating decision fatigue (Ceschi et al., 2017). On the other hand, stressors such as staff shortages and inadequate support consume more psychological resources, increase cognitive load when dealing with tasks, and exacerbate decision fatigue, thereby affecting work performance (Schweitzer et al., 2023). Therefore, the work environment directly and indirectly influences the degree of decision fatigue by affecting the distribution of stress and resources. Improving the work environment, providing sufficient support resources, and creating a positive work atmosphere are crucial for alleviating decision fatigue, enhancing nurses’ decision-making abilities, and improving overall work performance. Based on the above empirical evidence, Hypothesis H1 is proposed in this paper.

### 1.2 The mediating effect of job stress

Job stress arises from multiple factors, including workload and time allocation, patient care, work environment, and available resources. This stress leads to excessive depletion of nurses’ psychological resources as they cope with the demanding job requirements (Mirzaei et al., 2022; Norful et al., 2024). According to the Self-Control Theory (SCT) (Chew et al., 2021), an individual’s self-regulation resources are limited. Prolonged engagement in self-control behaviors (such as decision-making, attention control, and emotional regulation) results in the overconsumption of these resources, which in turn affects cognition, emotions, and behavior, leading to irrational decision-making by nurses. Research has confirmed that the proportion of conservative decisions made by nurses when referring patients increases by an average of 20.5% per hour between breaks (Dai et al., 2015). This phenomenon suggests that nurses who work under high-intensity conditions for extended periods are more likely to experience cognitive and emotional resource depletion, thereby affecting decision quality. Recent evidence from online mental-stress research supports this pattern: using NLP and sentiment analysis on Reddit data, Tuan et al. (2024) found that highly stressed individuals express more negative emotions and shift their communication patterns, underscoring how stress disrupts cognition, emotion, and behavior. However, despite these insights, there is still a lack of in-depth research on the mechanisms through which work-related stress specifically influences decision fatigue. Therefore, hypothesis H2 is proposed in this paper.

### 1.3 Resilience as a moderator in the first half of the mediating path

Resilience, as a positive adaptation process, refers to an individual’s ability to recover quickly from stress when faced with adversity. Studies have shown that improving nurses’ psychological resilience helps alleviate burnout and better cope with workplace stress (McFarland and Hlubocky, 2021; Rink et al., 2023). According to the triadic reciprocal determinism theory, individual behavior is shaped by a dynamic interaction of personal traits, environmental conditions, and their interplay (Ou et al., 2018). Personal resilience plays a crucial role in nurses’ decisions to stay in the profession and is strongly associated with their job satisfaction (Son and Ham, 2020). Nurses with high psychological resilience not only possess better emotional regulation and adaptability but are also able to effectively manage the stress of complex medical environments, aiding their recovery from adversity. Nurses with higher resilience are more proactive in making urgent medical decisions, can promptly identify signs of decision fatigue, and adjust their behavior to reduce its occurrence. In contrast, nurses with lower resilience are more susceptible to the negative impact of environmental stressors, which indirectly exacerbates decision fatigue. Therefore, hypothesis H3 is proposed in this paper.

## 2 Theoretical framework

The Job Demands–Resources (JD-R) model is a widely recognized framework for explaining occupational stress and its impact on employee health (Bakker and Demerouti, 2017). The model distinguishes between job demands, such as physical, psychological, and organizational pressure and job resources, which include material, social, and organizational support that help individuals manage these demands. In addition, personal resources play a protective role by buffering the adverse effects of high job demands and reducing stress-related outcomes.

**Figure 1:**
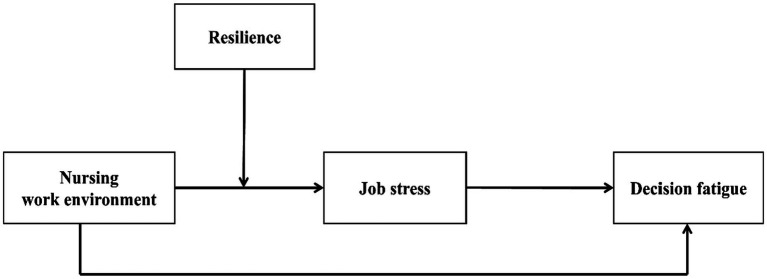
Hypothesized framework.

The Job Demands-Resources (JD-R) model serves as the guiding theoretical framework for this study, examines how job demands (job stress), job resources (nursing work environment), and personal resources (resilience) jointly influence nurses decision fatigue. Nurses typically encounter substantial job demands and inconsistent access to resources, placing them at heightened risk for decision fatigue, a form of mental exhaustion that impairs decision-making capacity (Middleton et al., 2021). Accordingly, this study investigates the interactive effects of job stress, the work environment, and psychological resilience on decision fatigue. Based on the JD-R framework, we formulated hypotheses and tested them using a moderated mediation model (see Figure 1).

### 2.1 Model hypotheses

*H1*: The nursing work environment has a significant negative effect on decision fatigue among nurses.

*H2*: Job stress mediates the relationship between the nursing work environment and decision fatigue.

*H3*: Resilience plays a moderating role in the first half of the mediating path.

## 3 Materials and methods

### 3.1 Study design

This study employed a cross-sectional design and followed the Strengthening the Reporting of Observational Studies in Epidemiology (STROBE) guidelines for cross-sectional studies.

### 3.2 Participants

This study adopted the convenience sampling method, which is a commonly used approach in clinical nursing research. From May 2023 to July 2023, clinical nurses from four public tertiary hospitals in Guangdong Province, China, were selected as the research subjects. The use of this sampling method was primarily due to its practicality, especially under limited research resources and time constraints.

The inclusion criteria for nurses participating in the survey were as follows: (1) aged between 18 and 60 years; (2) with over 1 year of clinical work experience; (3) having obtained a practicing nurse certificate; and (4) providing informed consent and voluntary participation. The exclusion criteria were: (1) nurse interns; (2) nurses undertaking training courses; and (3) nurses on maternity or sick leave during the survey period.

During data collection, two researchers reviewed each questionnaire to ensure its completeness and logical consistency. A total of 88 questionnaires were excluded because they had identical answers across all options or exhibited obvious logical inconsistencies. The number of valid questionnaires incorporated into the analysis was 812, resulting in a valid response rate of 90.2%.

### 3.3 Sample size calculation

The sample size (n) was estimated using the following sample size calculation formula for cross-sectional studies (Castro Alves and Kendall, 2018).

$n=\frac{Ζ\alpha^{2}*\sigma^{2}}{\delta^{2}}$

According to previous study, α =0.05, Zα^2^ =1.96,σ =6.0 is selected (Xie et al., 2022). It is expected that the allowable error does not exceed 0.5, adding a non-response rate of 20%. Finally, substituting into the formula, at least 693 nurses must be included. The sample size in this study exceeds the minimum sample size calculated.

### 3.4 Materials

#### 3.4.1 Demographic information

Based on previous literature (Pignatiello et al., 2020; Fernández-Miranda et al., 2023; Schweitzer et al., 2023) and refined through team discussions, we collected data on 13 key variables. These captured core demographic information (gender, age, marital status and educational level) and work-related factors (including hospital labor relations, number of children, work experience, professional title, average number of days off per month, night shift duty, personal monthly income (post-tax), number of patients responsible, and sleep quality).

#### 3.4.2 Decision fatigue scale

The Decision Fatigue Scale (DFS) was developed by Hickman et al. (2018). This scale was translated into Chinese and tested for reliability and validity by Pan and Li (2020), with a Cronbach’s *α* coefficient of 0.933 and a test–retest reliability of 0.838. The scale consists of 9 items and uses a 4-point Likert-type scale, with ratings ranging from 0 = strongly disagree to 3 strongly agree, and a higher score indicates a higher levels of decision fatigue. The Cronbach’s α coefficient of the scale in the present study was 0.942.

#### 3.4.3 Nursing work environment scale

The Nursing Work Environment Scale (NWES) was developed by Ye et al. (2016), was used to measure the nursing work environment. The scale includes 26 items divided into 7 dimensions:career and development, leadership and management, medical care relationships, recognition atmosphere, professional autonomy, basic guarantees, and adequate staffing. Items are scored using a 6-point Likert scale 1 = strongly disagree to 6 = strongly agree. A higher score indicates the better the nursing work environment, and the more it meets the nurses’ needs for the work environment. In this study, the Cronbach’s *α* coefficient of this scale was 0.974.

#### 3.4.4 Job stress scale

The Job Stress Scale was developed by Li and Liu (2000), was used to measure level of job stress experienced among nurses. The scale consists of 35 items divided into 5 dimensions, scored using a 4-point Likert scale 1 = no pressure to 4 = higher pressure.

The higher score reflect greater job stress levels. The Cronbach’s α coefficient of this research was 0.975.

#### 3.4.5 Resilience scale

The10-item Connor-Davidson Resilience Scale (CD-RISC-10) was initially developed by Campbell-Sills and Stein (2007), Connor and Davidson (2003), and it was translated into Chinese by Ye et al. (2017), with a Cronbach’s α coefficient of 0.851. The scale consists of 10 items and uses a 5-point Likert-type scale, with ratings ranging from 0 = never to 4 = always. The higher score indicates greater resilience levels. The Cronbach’s α coefficient of the scale was 0.965.

### 3.5 Data collection

In this study, data were collected online through the SoJump platform. Two researchers distributed the questionnaire link via this platform, and the general team leader coordinated with the directors of the hospital nursing departments to explain the survey objectives, subjects, and questionnaire completion methods. After obtaining their permission, the QR code and questionnaire link were sent to each hospital’s nursing department director via WeChat. On the first page of the online questionnaire, clear instructions were provided to explain the research purpose, significance, and filling instructions, emphasizing the principles of voluntariness, anonymity, and informed consent. Participants were required to actively indicate their consent by selecting between two options: “I agree to participate” and “I do not agree.” The survey platform was configured so that only those who selected “I agree” were redirected to the actual questionnaire items, while those who selected “I do not agree” were automatically directed to an exit page. This procedure ensured that informed consent was explicitly obtained from all participants. Subjects who agreed to participate were able to complete the questionnaire in approximately 8 min.

During the design process, all items in the questionnaire were made mandatory. If any responses were missing or incorrect, a prompt would appear after submission, requiring the participant to correct and resubmit the form before it could be successfully submitted. This ensured the completeness of the responses. To prevent duplicate submissions, the system was configured to allow each account and IP address to submit the questionnaire only once.

## 4 Data analysis

All statistical analyses were performed using SPSS 27.0 and PROCESS macro version 3.5.

First, descriptive statistics were used to characterize the sample, and independent t-tests and one-way ANOVA were applied to assess group differences in demographic factors related to decision fatigue. Second, Harman’s single-factor test were conducted to assess potential common method variance (CMV). Third, Pearson correlation analyses examined relationships among key variables. Finally, all key variables were standardized prior to testing mediation and moderation effects, to avoid multicollinearity and minimize bias in moderation analysis.

To test the hypotheses, mediation effects were analyzed using PROCESS Model 4 and moderated mediation effects were examined with Model 7 (Hayes, 2017). The 95% confidence intervals (CIs) were calculated using the bias-corrected percentile Bootstrap method with 5,000 resamples. If the range of the 95% CI did not include zero,it indicated the models were established. The significance level for all tests was set at *α* = 0.05. Both models controlled for covariates including hospital labor relations, number of children, personal monthly income(post-tax), and sleep quality, which were selected based on statistically significant associations (*p* < 0.05) in demographic analyses.

## 5 Results

### 5.1 Test of common method deviation

All data in this study were collected through the SoJump platform. We performed the Harman’s single-factor test to assess common method bias before data analysis. The results revealed that 10 factors with eigenvalues greater than 1 were extracted, and the explanatory variance of the first factor was less than 40%, which is below the judgment criterion (Zeng and Tan, 2021). This indicates that no common method bias was present in this study.

### 5.2 Characteristics of the participants

Participants were aged between 21 and 57 years. Male and female participants accounted for 3.8% and 96.2%, respectively. Clinical work experience ranged from 6 to 15 years (52%). Additionally, 67.6% of participants were married, and 81.3% held a bachelor’s degree. Table 1 provides additional detailed information about the nurses’ demographic characteristics.

**Table 1:** Difference of demographic factors in decision fatigue (*N* = 812).

Variables	M ± SD	*n* (%)	*t/F*	*P*
Gender			0.22	0.820
Male	11.29 ± 6.30	31(3.8)		
Female	11.05 ± 5.71	781(96.2)		
Age			0.79	0.497
≤25	11.62 ± 6.10	115(14.2)		
26 ~ 35	11.06 ± 5.98	434(53.4)		
36 ~ 45	10.73 ± 4.97	206(25.4)		
≥45	10.21 ± 5.63	57(7.0)		
Marital status			1.65	0.192
Unmarried	11.61 ± 5.51	245(30.2)		
Married	10.81 ± 5.82	549(67.6)		
Divorced/Widowed	10.88 ± 5.51	18 (2.2)		
Educational level			0.75	0.453
College or below	11.37 ± 5.5	152(18.7)		
Bachelor’s degree or above	10.98 ± 5.78	660(81.3)		
Work experience (years)			1.76	0.171
1 ~ 5	11.46 ± 5.16	177(21.8)		
6 ~ 15	11.19 ± 5.94	422(52.0)		
>15	10.45 ± 5.73	213(26.2)		
Professional title			1.73	0.176
Junior title	11.13 ± 5.83	462(56.9)		
Intermediate title	11.20 ± 5.65	293(36.1)		
Senior professional title	9.70 ± 5.17	57(7.0)		
Hospital labor relations			6.12	0.002
Authorized strength	10.54 ± 5.80	354(43.6)		
Contract employee	11.28 ± 5.60	431(53.1)		
Labor dispatch	5.66 ± 1.09	27(3.3)		
Number of children			3.55	0.029
0	7.24 ± 4.66	245(30.2)		
1	9.45 ± 4.68	230(28.3)		
≥2	14.93 ± 4.59	337(41.5)		
Average number of days off per month			0.97	0.329
<8	11.27 ± 5.89	372(45.8)		
≥8	10.87 ± 5.59	440(54.2)		
Whether night shift			−0.97	0.444
No	10.27 ± 5.66	142(17.5)		
Yes	11.13 ± 5.74	670(82.5)		
Personal monthly income (post-tax)			5.00	0.007
<7,000	12.34 ± 6.24	130(16.0)		
7,000 ~ 10,000	11.06 ± 5.88	377(46.4)		
≥10,000	10.50 ± 5.22	305(37.6)		
Number of patients responsible			2.39	0.092
≤6	10.52 ± 5.12	312(38.4)		
7 ~ 12	11.56 ± 5.85	236(29.1)		
≥12	11.23 ± 6.25	264(32.5)		
Sleep quality			32.13	<0.001
Good	8.24 ± 5.38	140(17.2)		
General	10.98 ± 5.22	453(55.8)		
Not good	13.02 ± 6.18	219(27.0)		
Job stress	86.08 ± 23.82	812	5.52	<0.001
Nursing work environment	107.88 ± 24.00	812	3.31	<0.001
Resilience	23.55 ± 8.32	812	3.13	<0.001

M, Mean; SD, Standard deviation; F, one-way ANOVA; *t* = independent *t*-test.

### 5.3 Correlation analysis of research variables

The strength of relationships was categorized as follows: weak (|r| < 0.3), moderate (0.3 ≤ |r| < 0.5), and strong (|r| ≥ 0.5), as outlined by Schober et al. (2018). A weak negative correlation was observed between decision fatigue and resilience (r = −0.224, *p* < 0.001). A moderate negative correlation was identified between decision fatigue and the nursing work environment (r = −0.367, *p* < 0.001). A strong positive correlation was found between decision fatigue and job stress (r = 0.572, *p* < 0.001) (see Table 2).

**Table 2:** Correlation coefficients of the study variables.

	Decision fatigue	Nursing working environment	Job stress	Resilience
Decision fatigue	1			
Nursing working environment	−0.367**	1		
Job stress	0.572**	−0.434**	1	
Resilience	−0.224	0.476	−0.049**	1

**p < 0.001.

### 5.4 Mediation effect of job stress on the relationship between nursing work environment and decision fatigue

In this study, relevant variables such as hospital labor relations, number of children, personal monthly income (post-tax), and sleep quality were controlled. Model 4 from Hayes’ PROCESS program was then used to examine the mediating effect of job stress between the nursing work environment and decision fatigue.

The results of the mediation effect analysis showed that the nursing work environment negatively predicted decision fatigue (*β* = −0.040, *p* = 0.034). The nursing work environment had a significant negative effect on job stress (β = −0.260, *p* < 0.001). Job stress was a significant positive predictor of decision fatigue (β = 0.081, *p* < 0.003) (see Table 3). The mediation effect of job stress was confirmed with a 95% confidence interval (CI) that did not include 0 (Bootstrap 95% CI = [−0.035, −0.008]). The direct effect of the independent variable on the dependent variable was −0.041, while the mediating effect was −0.021 (see Table 4). These effects accounted for 66.1% and 33.9% of the total effect (−0.062), respectively, indicating that the nursing work environment influences decision fatigue partially through the mediation of job stress.

**Table 3:** Standardised estimation of each path in the mediation model.

Path	Coefficient			Model summary		
	β	SE	*P*	*R* ^2^	*F*	*P*
Model 1						
Nursing work environment → job stress	−0.260	0.029	<0.001	0.640	112.349	<0.001
Model 2						
Nursing work environment → decision fatigue	−0.062	0.018	0.009	0.873	515.463	<0.001
Model 3						
Nursing work environment → decision fatigue	−0.040	0.019	0.034	0.875	438.361	<0.001
Job stress → decision fatigue	0.081	0.022	0.003			

**Table 4:** Testing the mediation effect of job stress on decision fatigue.

Type	Effect	Boot SE	LLCI	ULCI	Effect size (%)
Total effect	−0.062	0.018	−0.098	−0.026	100
Direct effect	−0.041	0.019	−0.078	−0.003	66.1
Indirect effect	−0.021	0.007	−0.035	−0.008	33.9

Boot. LLCI, the lower limit confidence interval of effects estimated by Bootstrap Method; Boot. SE, the standard error of effects estimated by Bootstrap method; Boot. ULCI, the upper limit confidence interval of effects estimated by Bootstrap method.

### 5.5 Moderated effect of resilience the mediation model

**Figure 2:**
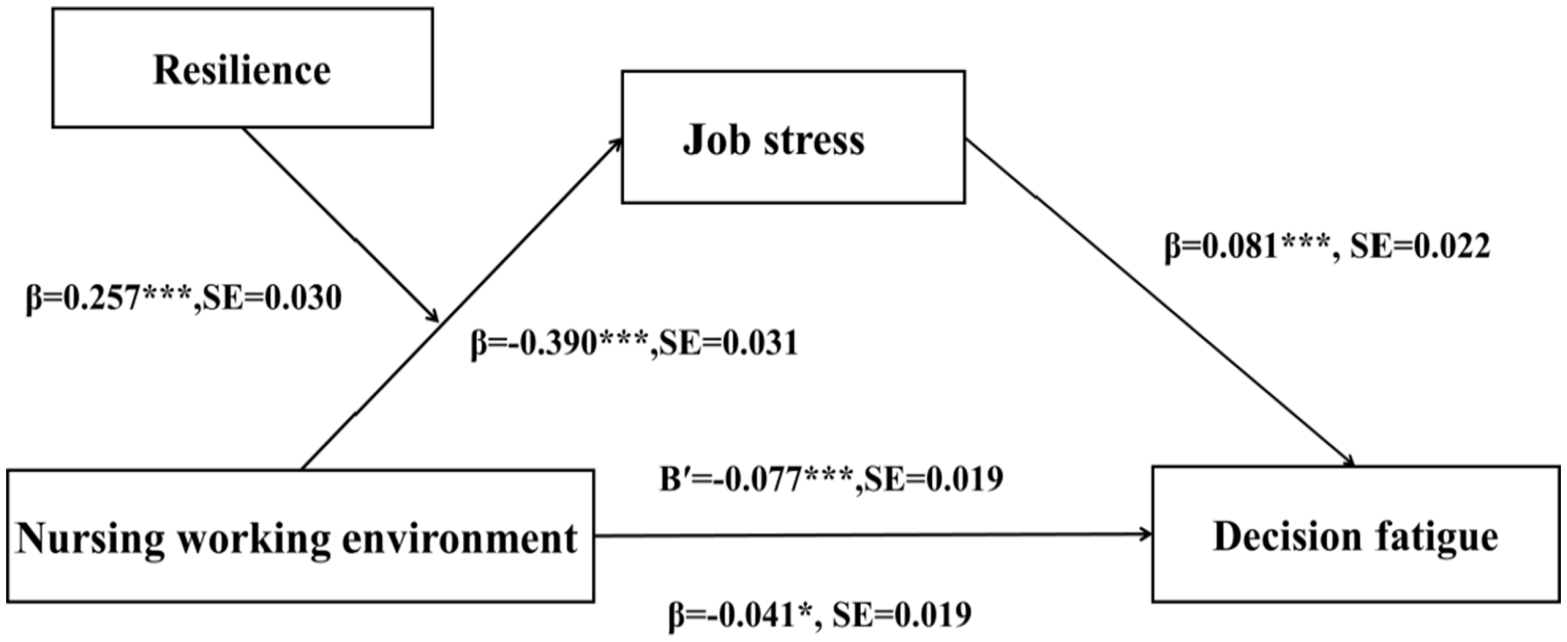
Representation of the mediation effect of job stress (moderated by resilience) on the relationship between nursing work environment and decision fatigue. B′, Nursing work environment* Resilience; *p < 0.05; ***p < 0.001.

**Figure 3:**
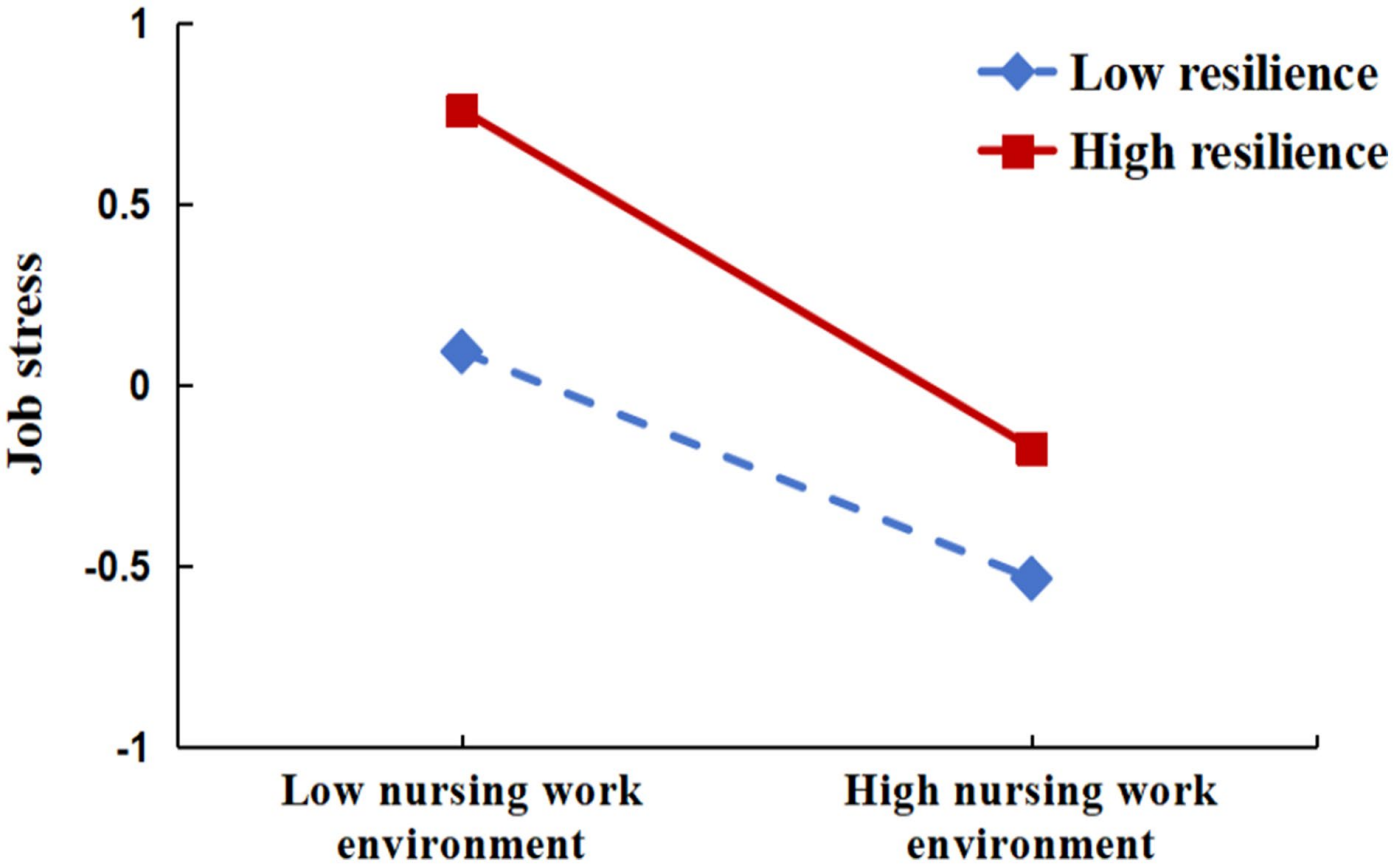
The moderated effect of resilience on the relationship between the nursing work environment and job stress.

To test the moderating role of the nursing work environment in the mediation path, this study utilized Model 7 in PROCESS (Hayes, 2017) to conduct a conditional process analysis, based on 5,000 bootstrap repetitions for self-sampling. The results revealed that resilience moderates the indirect effect of the nursing work environment on decision fatigue through job stress (*β* = −0.077, t = −4.059, *p* < 0.001). Specifically, resilience significantly influences the extent to which job stress mediates the relationship between the nursing work environment and decision fatigue among clinical nurses (see Table 5 and Figure 2). These findings provide support for the hypothesis that resilience moderates the indirect effect of the nursing work environment on decision fatigue through job stress. Table 5 presents the significance of the indirect effect of the nursing work environment on decision fatigue via job stress at three different levels of resilience. At higher levels of resilience, the indirect effect on decision fatigue was significant (β = −0.467, SE = 0.039, 95% CI [−0.544, −0.390]), with the confidence interval excluding 0. Similarly, at lower levels of resilience, the indirect effect remained significant (β = −0.314, SE = 0.033, 95% CI [−0.379, −0.248]), with the confidence interval not containing 0. A simple slope analysis further revealed that job stress significantly predicted decision fatigue, irrespective of resilience levels. As shown in Figure 3, compared to nurses with high resilience, the influence of the nursing work environment on job stress was greater for nurses with low resilience. Moreover, the impact of the nursing work environment on job stress increased as resilience levels decreased.

**Table 5:** The moderated mediating impact of resilience through the nursing work environment and job stress.

Model	*β*	SE	*t*	*P*	LLCI	ULCI
Mediation model analysis: job stress as the outcome variable						
Constant	0.463	0.217	2.133	0.033	0.037	0.889
Hospital labor relations	0.081	0.047	1.732	0.084	−0.011	0.172
Number of children	0.226	0.051	4.463	<0.001	0.127	0.325
Personal monthly income (post-tax)	−0.462	0.059	−7.814	<0.001	−0.578	−0.346
Sleep quality	0.103	0.041	2.513	0.012	0.023	0.183
Nursing work environment (NWE)	−0.390	0.031	−12.510	<0.001	−0.452	−0.330
Resilience	0.257	0.030	8.679	<0.001	0.199	0.316
NWE × Resilience	−0.077	0.019	−4.059	<0.001	−0.114	−0.040
*R* ^2^	0.683					
*F*	100.503					
*P*	<0.001					
Dependent model analysis: decision fatigue as the outcome variable						
Constant	0.003	0.144	0.022	0.982	−0.279	0.285
Hospital labor relations	−0.026	0.031	−0.851	0.395	−0.087	0.034
Number of children	0.617	0.034	18.392	<0.001	0.551	0.683
Personal monthly income (post-tax)	−0.430	0.041	−10.612	<0.001	−0.509	−0.350
Sleep quality	0.145	0.027	5.336	<0.001	0.092	0.198
Nursing work environment (NWE)	−0.041	0.019	−2.126	0.033	−0.078	−0.003
Job stress	0.081	0.022	3.654	<0.001	0.038	0.125
*R* ^2^	0.875					
*F*	438.362					
*P*	<0.001					
Indirect effect model						
NWE → Job stress → Decision fatigue	−0.032	0.010	−3.235	<0.001	−0.051	−0.013

Conditional indirect effects of resilience						
Resilience	Effect	SE	*t*	*P*	LLCI	ULCI
Low resilience	−0.314	0.033	−9.412	<0.001	−0.379	−0.248
Resilience means	−0.390	0.031	−12.510	<0.001	−0.452	−0.329
High resilience	−0.467	0.039	−11.863	<0.001	−0.544	−0.390

β, effect; SE, the standard error of indirect effects estimated; Index, index of moderated mediation model; LLCI, lower limit confidence interval; ULCI, upper limit confidence interval.

## 6 Discussion

### 6.1 The association between nursing work environment and decision fatigue

The results of this study indicate that the nursing work environment has a significant negative impact on decision fatigue, supporting Hypothesis 1. This finding suggests that nurses’ perception of a positive work environment can help alleviate cognitive and emotional burdens, thereby reducing the occurrence of decision fatigue. This aligns with the conclusions of (Pignatiello et al., 2022; Wei et al., 2018). We have confirmed that a better nursing work environment is negatively associated with decision fatigue. Clinical decision-making involves a complex critical thinking process, including a careful assessment of the patient’s history and the application of both theoretical and experiential knowledge (Nibbelink and Brewer, 2018; Thompson et al., 2013; Pignatiello et al., 2022). Research has shown that in high-intensity work environments, nurses often face an overload of decision-making tasks, leading to decision fatigue (Fernandez et al., 2020; Kiptulon et al., 2024). Over time, decision fatigue can impair a nurse’s ability to process relevant information quickly and to make and implement effective decisions (Natal and Saltzman, 2022). The findings also suggest that a poor nursing work environment can reduce job satisfaction, increase professional burnout, and elevate turnover risks, making it difficult to establish strong connections with the organization (Poku et al., 2022). These findings also indicate that improving the work environment could reduce decision fatigue and enhance both the physical and mental health of nurses. Therefore, nursing managers should prioritize creating a supportive work atmosphere by allocating sufficient human resources. This approach can help alleviate negative emotions, improve the overall environmental quality, and reduce nurses’ decision fatigue.

### 6.2 The mediating role of job stress between the nursing work environment and decision fatigue

This study confirmed that job stress acts as a mediating mechanism between the nursing work environment and decision fatigue, demonstrating both direct and indirect effects, which supports Hypothesis 2. The work environment influences decision fatigue directly and indirectly through job stress. Nurses who experience higher stress levels report significantly greater decision fatigue. A positive correlation emerged: the severity of decision fatigue increased with higher levels of job stress, with stress levels serving as a direct predictor of the incidence of fatigue. According to the job demands-resources model (JD-R), the high demands of the nursing profession, coupled with a lack of job resources, lead to an increase in job stress. Meanwhile, high-pressure clinical decision-making depletes nurses’ psychological and cognitive resources. Repeated complex choices lead to impaired decision-making ability, cognitive decline, and emotional dysfunction, often manifesting as avoidant behaviors (Heatherton and Wagner, 2011; Keykaleh et al., 2018; Dubash et al., 2020). In their daily practice, nurses are confronted with a range of patient needs that require frequent, high-stakes decisions. The ongoing process of decision-making leads to the accumulation of fatigue, which ultimately affects their physical and emotional well-being (Fernández-Miranda et al., 2023; Schweitzer et al., 2023). Previous studies have consistently shown (Allan et al., 2019; Rao and Nyquist, 2018) that decision-making behavior in rest and interruptions helps reduce the occurrence of decision fatigue. It is recommended that nursing managers place more emphasis on decision fatigue among nurses. Providing a supportive environment to alleviate high work pressure and decision fatigue requires proper management training, the provision of adequate equipment and manpower, as well as the development of resilient skills to help nurses manage stress. At the same time, adjusting nurses’ workload distribution and making reasonable rest arrangements to lower work intensity and decrease decision fatigue are crucial for promoting a sustainable nursing team.

### 6.3 The moderating role of resilience

The results of the moderating effect analysis indicate that resilience significantly moderates the first half of the mediating path. A negative correlation was observed between decision fatigue and resilience, suggesting that decision fatigue diminishes resilience over time. The JD-R model (Bakker and Demerouti, 2017) has been validated, with psychological resilience identified as a key personal resource influencing nurse decision fatigue. This finding further supports Hypothesis 3. The simple slope test indicated that a supportive nursing work environment reduces job stress more effectively when resilience levels are higher. At higher levels of mental toughness, nurses demonstrate greater self-regulation and adaptability, emphasizing its nuanced role in moderating stress responses (González-Siles et al., 2022). However, this effect may be contingent on factors such as personal traits and organizational support, necessitating further investigation. Nurses in low-support care environments reported the highest levels of job stress and decision fatigue, consistent with prior evidence that high-pressure settings necessitate rapid decision-making, thereby increasing error risks and depleting self-control motivation (Arslanian-Engoren and Scott, 2014; Tabakakis et al., 2019). To address this, targeted interventions to bolster resilience, such as group cognitive therapy, mindfulness-based stress reduction, and relaxation training, should be prioritized. In addition, digital interventions, including mobile health applications and virtual resilience training programs, represent feasible and scalable approaches for enhancing nurses’ resilience (Concilio et al., 2021). These technology-based tools can provide flexible, accessible, and individualized support, making them particularly suitable for nurses working under high workload conditions. Nursing managers are advised to assess resilience levels and foster supportive organizational environments to alleviate decision fatigue.

## 7 Limitations and future directions

The limitations of this study include: (1) This study adopted a cross-sectional design, which may restrict the ability to infer causal relationships. Future longitudinal studies could further explore the long-term effects and causal pathways among the variables. In addition, future research could incorporate mixed-methods approaches to gain a more comprehensive understanding by integrating quantitative findings with qualitative insights.(2) The tools used for data collection in this study were all self-reported scales, which inherently carry a degree of subjectivity and may lead to biased results. This suggests the need to combine qualitative research methods to explore the factors influencing decision fatigue among clinical nurses more deeply, providing a theoretical basis for further adjustment of intervention strategies.

## 8 Conclusion

This study findings suggest that the nursing work environment impacts decision fatigue through job stress, with resilience as a moderating factor between the nursing work environment and job stress. Hence, in addressing nurse decision fatigue, healthcare professionals may benefit from adopting a psychological perspective, recognizing the multifaceted influences of the work environment, and prioritizing the enhancement of psychological resilience.

Tailored strategies based on factors such as the nursing work environment and psychological conditions can effectively mitigate job stress and bolster psychological resilience, reducing the likelihood of decision fatigue. This is proposed to improve nurses’ work efficiency and psychological well-being, establishing a solid foundation for delivering superior nursing care.

## Data Availability

The original contributions presented in the study are included in the article, further inquiries can be directed to the corresponding author.

## References

[ref9006] Al-Akashee B. Barhoumi W. Algharbawi F. A. Hegazy F. (2024). Challenges of women healthcare workers during COVID-19. Emerg. Sci. J. 8, 2157–2171. doi: 10.28991/esj-2024-08-04-017

[ref1] Allan J. L. Johnston D. W. Powell D. J. H. Farquharson B. Jones M. C. Leckie G. . (2019). Clinical decisions and time since rest break: an analysis of decision fatigue in nurses. Health Psychol. 38, 318–324. doi: 10.1037/hea0000725, 30896218

[ref2] Arslanian-Engoren C. Scott L. D. (2014). Clinical decision regret among critical care nurses: a qualitative analysis. Heart Lung 43, 416–419. doi: 10.1016/j.hrtlng.2014.02.006, 24655941

[ref3] Ayres P. Lee J. Y. Paas F. van Merriënboer J. J. G. (2021). The validity of physiological measures to identify differences in intrinsic cognitive load. Front. Psychol. 12:702538. doi: 10.3389/fpsyg.2021.702538, 34566780 PMC8461231

[ref9005] Bakker A. B. Demerouti E. (2017). Job demands-resources theory: taking stock and looking forward. J. Occup. Health Psychol. 22, 273–285. doi: 10.1037/ocp0000056 27732008

[ref5] Baumeister R. F. Bratslavsky E. Muraven M. Tice D. M. (2018). Ego depletion: is the active self a limited resource? J. Pers. Soc. Psychol. 74, 1252–1265. doi: 10.1037/0022-3514.74.5.1252 9599441

[ref7] Bell T. Sprajcer M. Flenady T. Sahay A. (2023). Fatigue in nurses and medication administration errors: a scoping review. J. Clin. Nurs. 32, 5445–5460. doi: 10.1111/jocn.16620, 36707921

[ref8] Blissett S. Rodriguez S. Qasim A. O'Sullivan P. (2021). Learning echocardiography in the workplace: a cognitive load perspective. Acad. Med. 96, 441–448. doi: 10.1097/ACM.0000000000003789, 33031115

[ref9004] Cai Y. Chen Yii J. L. Pathak S. (2024). The effect of EAP on job performance based on psychological contract and perceived organizational support. Emerg. Sci. J. 8, 2172–2187. doi: 10.28991/esj-2024-08-05-018

[ref9] Campbell-Sills L. Stein M. B. (2007). Psychometric analysis and refinement of the Connor-davidson resilience scale (CD-RISC): validation of a 10-item measure of resilience. J. Trauma. Stress. 20, 1019–1028. doi: 10.1002/jts.20271, 18157881

[ref9007] Castro Alves L. Kendall M. C. (2018). Sample size and the establishment of safety in perioperative medicine. Actas Urol. Esp. 42, 610. doi: 10.1016/j.acuro.2018.05.007 30007777

[ref10] Ceschi A. Demerouti E. Sartori R. Weller J. (2017). Decision-making processes in the workplace: how exhaustion, lack of resources and job demands impair them and affect performance. Front. Psychol. 8:313. doi: 10.3389/fpsyg.2017.00313, 28529491 PMC5418353

[ref11] Chew H. S. J. Sim K. L. D. Choi K. C. Chair S. Y. (2021). Effectiveness of a nurse-led temporal self-regulation theory-based program on heart failure self-care: a randomized controlled trial. Int. J. Nurs. Stud. 115:103872. doi: 10.1016/j.ijnurstu.2021.103872, 33516047

[ref13] Concilio L. Lockhart J. S. Kronk R. Oermann M. Brannan J. Schreiber J. B. (2021). Impact of a digital intervention on perceived stress, resiliency, social support, and intention to leave among newly licensed graduate nurses: a randomized controlled trial. J. Contin. Educ. Nurs. 52, 367–374. doi: 10.3928/00220124-20210714-06, 34324377

[ref14] Connor K. M. Davidson J. R. (2003). Development of a new resilience scale: the Connor-Davidson resilience scale (CD-RISC). Depress. Anxiety 18, 76–82. doi: 10.1002/da.10113, 12964174

[ref15] Dai H. Milkman K. L. Hofmann D. A. Staats B. R. (2015). The impact of time at work and time off from work on rule compliance: the case of hand hygiene in health care. J. Appl. Psychol. 100, 846–862. doi: 10.1037/a0038067, 25365728

[ref16] Delgado C. Roche M. Fethney J. Foster K. (2021). Mental health nurses' psychological well-being, mental distress, and workplace resilience: a cross-sectional survey. Int. J. Ment. Health Nurs. 30, 1234–1247. doi: 10.1111/inm.12874, 33913226

[ref17] Dong S. S. Wang K. Zhang K. Q. Wang X. H. Wang J. H. Turdi S. . (2024). Decision fatigue experience of front-line nurses in the context of public health emergency: an interpretative phenomenological analysis. BMC Nurs. 23:553. doi: 10.1186/s12912-024-02163-w, 39135083 PMC11321180

[ref18] Dubash R. Bertenshaw C. Ho J. H. (2020). Decision fatigue in the emergency department. Emerg. Med. Australas. 32, 1059–1061. doi: 10.1111/1742-6723.13670, 33103832

[ref19] Falguera C. C. De Los Santos J. A. A. Galabay J. R. Firmo C. N. Tsaras K. Rosales R. A. . (2021). Relationship between nurse practice environment and work outcomes: a survey study in the Philippines. Int. J. Nurs. Pract. 27:e12873. doi: 10.1111/ijn.12873, 32677223

[ref20] Fernandez R. Lord H. Halcomb E. Moxham L. Middleton R. Alananzeh I. . (2020). Implications for COVID-19: a systematic review of nurses' experiences of working in acute care hospital settings during a respiratory pandemic. Int. J. Nurs. Stud. 111:103637. doi: 10.1016/j.ijnurstu.2020.103637, 32919358 PMC7206441

[ref21] Fernández-Miranda G. Urriago-Rayo J. Akle V. Noguera E. Mejía N. Amaya S. . (2023). Compassion and decision fatigue among healthcare workers during COVID-19 pandemic in a Colombian sample. PLoS One 18:e0282949. doi: 10.1371/journal.pone.0282949, 36961780 PMC10038311

[ref23] González-Siles P. Martí-Vilar M. González-Sala F. Merino-Soto C. Toledano-Toledano F. (2022). Sense of coherence and work stress or well-being in care professionals: a systematic review. Healthcare 10:1347. doi: 10.3390/healthcare10071347, 35885873 PMC9323122

[ref24] Gündüz E. S. Yildirim N. Akatin Y. Gündoğdu N. A. (2024). Relationship between nurses' resilience and quality of professional life. Int. Nurs. Rev. 71, 1023–1031. doi: 10.1111/inr.12960, 38511869

[ref26] Hayes A. F. (2017). Partial, conditional, and moderated moderated mediation: quantification, inference, and interpretation. Commun. Monogr. 85, 4–40. doi: 10.1080/03637751.2017.1352100

[ref27] Heatherton T. F. Wagner D. D. (2011). Cognitive neuroscience of self-regulation failure. Trends Cogn. Sci. 15, 132–139. doi: 10.1016/j.tics.2010.12.005, 21273114 PMC3062191

[ref28] Hickman R. L. Jr. Pignatiello G. A. Tahir S. (2018). Evaluation of the decisional fatigue scale among surrogate decision makers of the critically ill. West. J. Nurs. Res. 40, 191–208. doi: 10.1177/0193945917723828, 28805132 PMC5750078

[ref30] Keykaleh M. S. Safarpour H. Yousefian S. Faghisolouk F. Mohammadi E. Ghomian Z. (2018). The relationship between nurse's job stress and patient safety. Open Access Maced. J. Med. Sci. 6, 2228–2232. doi: 10.3889/oamjms.2018.351, 30559893 PMC6290432

[ref31] Kiptulon E. K. Elmadani M. Limungi G. M. Simon K. Tóth L. Horvath E. . (2024). Transforming nursing work environments: the impact of organizational culture on work-related stress among nurses: a systematic review. BMC Health Serv. Res. 24:1526. doi: 10.1186/s12913-024-12003-x, 39623348 PMC11613752

[ref32] Kohnen D. De Witte H. Schaufeli W. B. Dello S. Bruyneel L. Sermeus W. (2023). What makes nurses flourish at work? How the perceived clinical work environment relates to nurse motivation and well-being: a cross-sectional study. Int. J. Nurs. Stud. 148:104567. doi: 10.1016/j.ijnurstu.2023.104567, 37837704

[ref33] Lee S. E. Scott L. D. (2018). Hospital nurses' work environment characteristics and patient safety outcomes: a literature review. West. J. Nurs. Res. 40, 121–145. doi: 10.1177/0193945916666071, 27586440

[ref34] Li X. Liu Y. (2000). Job stressors and burnout among staff nurses. Chin. J. Nurs. 35, 645–649.

[ref35] McFarland D. C. Hlubocky F. (2021). Therapeutic strategies to tackle burnout and emotional exhaustion in frontline medical staff: narrative review. Psychol. Res. Behav. Manag. 14, 1429–1436. doi: 10.2147/PRBM.S256228, 34552358 PMC8450185

[ref36] Middleton R. Loveday C. Hobbs C. Almasi E. Moxham L. Green H. . (2021). The COVID-19 pandemic-a focus on nurse managers' mental health, coping behaviours and organisational commitment. Collegian 28, 703–708. doi: 10.1016/j.colegn.2021.10.006, 34744479 PMC8556582

[ref37] Mirzaei A. Mozaffari N. Habibi Soola A. (2022). Occupational stress and its relationship with spiritual coping among emergency department nurses and emergency medical services staff. Int. Emerg. Nurs. 62:101170. doi: 10.1016/j.ienj.2022.101170, 35487041 PMC9040637

[ref39] Natal G. Saltzman B. (2022). Decisions, decisions, decisions: decision fatigue in academic librarianship. J. Acad. Librariansh. 48:102476. doi: 10.1016/j.acalib.2021.102476

[ref40] Nibbelink C. W. Brewer B. B. (2018). Decision-making in nursing practice: an integrative literature review. J. Clin. Nurs. 27, 917–928. doi: 10.1111/jocn.14151, 29098746 PMC5867219

[ref41] Norful A. A. Albloushi M. Zhao J. Gao Y. Castro J. Palaganas E. . (2024). Modifiable work stress factors and psychological health risk among nurses working within 13 countries. J. Nurs. Scholarsh. 56, 742–751. doi: 10.1111/jnu.12994, 38816945 PMC11530301

[ref42] Ou H. Chen J. C. Castro-Alonso J. C. Fred P. John S. (2018). Extending cognitive load theory to incorporate working memory resource depletion: evidence from the spacing effect. Educ. Psychol. Rev. 30, 483–501. doi: 10.1007/s10648-017-9426-2

[ref43] Pan G. Li M. (2020). Translation and validation of the Chinese version of the decision fatigue scale applied to ICU patient family members. J. Nurs. 27, 38–41. doi: 10.16460/j.issn1008-9969.2020.12.038

[ref44] Persson E. Barrafrem K. Meunier A. Tinghög G. (2019). The effect of decision fatigue on surgeons' clinical decision making. Health Econ. 28, 1194–1203. doi: 10.1002/hec.3933, 31344303 PMC6851887

[ref45] Pignatiello G. A. Martin R. J. Hickman R. L. Jr. (2020). Decision fatigue: a conceptual analysis. J. Health Psychol. 25, 123–135. doi: 10.1177/1359105318763510, 29569950 PMC6119549

[ref46] Pignatiello G. A. Tsivitse E. O'Brien J. Kraus N. Hickman R. L. Jr. (2022). Decision fatigue among clinical nurses during the COVID-19 pandemic. J. Clin. Nurs. 31, 869–877. doi: 10.1111/jocn.15939, 34291521 PMC8447365

[ref47] Poku C. A. Donkor E. Naab F. (2022). Impacts of nursing work environment on turnover intentions: the mediating role of burnout in Ghana. Nurs. Res. Pract. 2022, 1–9. doi: 10.1155/2022/1310508, 35265373 PMC8898860

[ref48] Rao S. Nyquist A. C. (2018). The power of the nudge to decrease decision fatigue and increase influenza vaccination rates. JAMA Netw. Open 1:e181754. doi: 10.1001/jamanetworkopen.2018.1754, 30646149

[ref49] Ren H. Li P. Xue Y. Xin W. Yin X. Li H. (2024). Global prevalence of nurse turnover rates: a Meta-analysis of 21 studies from 14 countries. J. Nurs. Manag. 2024:5063998. doi: 10.1155/2024/5063998, 40224762 PMC11919231

[ref50] Rink L. C. Silva S. G. Adair K. C. Oyesanya T. O. Humphreys J. C. Sexton J. B. (2023). Characterizing burnout and resilience among nurses: a latent profile analysis of emotional exhaustion, emotional thriving and emotional recovery. Nurs. Open 10, 7279–7291. doi: 10.1002/nop2.1980, 37661657 PMC10563410

[ref51] Schweitzer D. R. Baumeister R. Laakso E. L. Ting J. (2023). Self-control, limited willpower and decision fatigue in healthcare settings. Intern. Med. J. 53, 1076–1080. doi: 10.1111/imj.16121, 37294047

[ref9001] Schober P. Boer C. Schwarte L. A. (2018). Correlation coefficients: appropriate use and interpretation. Anesth. Analg. 126, 1763–1768. doi: 10.1213/ANE.0000000000002864 29481436

[ref52] Scott L. D. Arslanian-Engoren C. Engoren M. C. (2014). Association of sleep and fatigue with decision regret among critical care nurses. Am. J. Crit. Care 23, 13–23. doi: 10.4037/ajcc2014191, 24382613

[ref53] Son D. M. Ham O. K. (2020). Influence of group resilience on job satisfaction among Korean nurses: a cross-sectional study. J. Clin. Nurs. 29, 3473–3481. doi: 10.1111/jocn.15385, 32562557

[ref54] Tabakakis C. K. McAllister M. Bradshaw J. To, Q. G (2019). Psychological resilience in New Zealand registered nurses: the role of workplace characteristics. J. Nurs. Manag. 27, 1351–1358. doi: 10.1111/jonm.12815 31220386

[ref9003] Thompson C. Cullum N. McCaughan D. Sheldon T. Raynor P. (2013). An agenda for clinical decision making and judgement in nursing research and education. Int. J. Nurs. Stud. 50, 1720–1726. doi: 10.1016/j.ijnurstu.2013.07.002 23747201

[ref9002] Tuan T. A. Nghia N. H. An T. D. Loan D. T. T. (2024). Exploring mental stress expressions in online communities: a subreddit analysis. J. Hum. Earth. Future 2024. doi: 10.28991/hef-2024-05-02-01

[ref55] Wei H. Sewell K. A. Woody G. Rose M. A. (2018). The state of the science of nurse work environments in the United States: a systematic review. Int. J. Nurs. Sci. 5, 287–300. doi: 10.1016/j.ijnss.2018.04.010, 31406839 PMC6626229

[ref56] Wilby K. J. Paravattil B. (2021). Cognitive load theory: implications for assessment in pharmacy education. Res. Social Adm. Pharm. 17, 1645–1649. doi: 10.1016/j.sapharm.2020.12.009, 33358136

[ref57] Xie W. Sun G. Gao M. Wen G. (2022). Status quo and correlation analysis of decision making fatigue and occupational well-being among nurses in cardiovascular medicine. Chin. Med. Herb. 19, 43–46. doi: 10.20047/j.issn1673-7210.2022.26.09

[ref58] Ye Z. J. Qiu H. Z. Li P. F. Chen P. Liang M. Z. Liu M. L. . (2017). Validation and application of the Chinese version of the 10-item Connor-Davidson resilience scale (CD-RISC-10) among parents of children with cancer diagnosis. Eur. J. Oncol. Nurs. 27, 36–44. doi: 10.1016/j.ejon.2017.01.004, 28279394

[ref59] Ye Z. Shao J. Tang L. (2016). The development of the nursing work environment scale and its reliability and validity. Chin. J. Nurs. 51, 142–147. doi: 10.3761/j.issn.0254-1769.2016.02.002

[ref60] Zeng X. Tan C. (2021). The relationship between the family functioning of individuals with drug addiction and relapse tendency: a moderated mediation model. Int. J. Environ. Res. Public Health 18:625. doi: 10.3390/ijerph18020625, 33451020 PMC7828550

[ref61] Zhu Y. Zhang Y. Wang X. Fan X. Deng X. Li L. (2021). Decision fatigue among medical staff: a review. J. Nurs. Sci. 36, 101–104. doi: 10.3870/j.issn.1001-4152.2021.01.101

